# Migrants’ mental health recovery in Italian reception facilities

**DOI:** 10.1038/s43856-023-00385-8

**Published:** 2023-11-22

**Authors:** Emanuele Caroppo, Carmela Calabrese, Marianna Mazza, Alessandro Rinaldi, Daniele Coluzzi, Pierangela Napoli, Martina Sapienza, UOC Salute Mentale working group, Italo Monfrinotti, Michela Bosio, Francesco Colosimo, Francesco Rita, Fabrizio Perrelli, Annalisa Rosso, Maurizio Porfiri, Pietro De Lellis

**Affiliations:** 1 Department of Mental Health, Local Health Authority Roma 2, Rome, Italy; 2 Department of Electrical Engineering and Information Technology, University of Naples Federico II, Naples, Italy; 3 Institut de Neurosciences des Systémes (INS), Aix Marseille Université, 13 Marseille, France; 4 Institute of Psychiatry and Psychology, Department of Geriatrics, Neuroscience and Orthopedics, Fondazione Policlinico Universitario A. Gemelli IRCCS, Università Cattolica del Sacro Cuore, Rome, Italy; 5 Department of Psychiatry, Università Cattolica del Sacro Cuore, Rome, Italy; 6 Migrant Health Unit, Local Health Authority Roma 2, Rome, Italy; 7 Department of Life Sciences and Public Health, Università Cattolica del Sacro Cuore, Rome, Italy; 8 Center for Urban Science and Progress, Department of Mechanical and Aerospace Engineering, and Department of Biomedical Engineering, New York University Tandon School of Engineering, Brooklyn, NY USA

**Keywords:** Population screening, Post-traumatic stress disorder

## Abstract

**Background:**

Forced migration leaves deep marks on the psychological well-being of migrants, with post-traumatic stress disorder (PTSD) and other psychological conditions being prevalent among them. While research has clarified the extent to which pre-migration trauma is a predictor of mental health outcomes, the role of post-migration stressors in the settlement environment are yet to be fully characterized.

**Methods:**

We monitored mental health of a cohort of 100 asylum-seekers during their 14-day COVID-19-related quarantine in reception facilities in Rome, Italy, through the administration of six questionnaires (a demographic survey, the WHO-5 well-being index, the Primary Care PTSD Screen for Diagnostic and Statistical Manual of Mental Disorders 5 (DSM-5), the Harvard Trauma Questionnaire, the Trauma and Loss Spectrum—Self Report, and the LiMEs—Italian version). Through the combination of statistical analysis and supervised learning, we studied the impact of the first contact with the reception system on asylum-seekers’ mental health and sought for possible risk and shielding factors for PTSD.

**Results:**

We find that sheltering in refugee centers has a positive impact on migrants’ mental health; asylum-seekers with PTSD reported more traumatic events and personality characteristics related to loss and trauma; life events are predictors of PTSD in asylum-seekers.

**Conclusions:**

We identify past traumatic experiences as predictors of PTSD, and establish the positive role the immediate post-migration environment can play on migrants’ psychological well-being. We recommend for host countries to implement reception models that provide effective protection and integration of asylum-seekers, similar to those in the Italian system.

## Plain Language Summary

Traumatic experiences before and during migration can impact the psychological well-being of migrants. This can result in post-traumatic stress disorder (PTSD), a disorder in which prior experience of traumatic events can lead to severe anxiety. We asked migrants to fill in questionnaires about their well-being during a 14-day quarantine in reception facilities in Rome, Italy. We evaluated whether the migrants had symptoms of PTSD and the impact of the quarantine on their mental health. We found that migrants who had experienced past traumatic events were more likely to have PTSD, and that staying in a safe and welcoming place in Italy helped improve their mental wellbeing. These findings underline the importance of designing suitable policies to support migrants’ mental health when they arrive in host countries.

## Abstract (web summary)

Caroppo, Calabrese et al. monitor the mental health of 100 asylum-seekers during a 14-day COVID19 related quarantine in reception facilities in Rome, Italy. Asylum-seekers are more likely to experience post-traumatic stress disorder than the general population, but a supportive environment during quarantine enhances their psychological well-being.

## Introduction

Human migration is a universal phenomenon that has characterized the humanity throughout its journey on this planet^1^. The reasons why humans migrate can be very diverse, but what they have in common is the search for better living conditions. Migrants may decide to relocate for economical or aspirational reasons, or be forced to migrate as consequence of natural disasters^2^, climate change^3^, war^4^, or political instability^5^. International migrations have been steadily increasing over the last decades, reaching a total of 281 million people (3.6% of the world population) living in a country other than their country of birth by 2020^6^.

The migration process has an impact on the health of the migrant population, causing physical and mental harm. In forced migrations, several factors, such as transport in closed containers, accidental injuries, malnutrition, and accommodation in detention centers and refugee camps influence the physical health of refugees. The most prevalent infections in refugees and asylum-seekers include tuberculosis and other respiratory tract infections, parasitic diseases, and intestinal parasitic infections. Further, migrants are exposed to important risk factors of disparate diseases, which include anemia, hyperlipidemia, arterial hypertension, diabetes, smoking, overweight, and malnutrition^7^. Even when migration is voluntary and migrants achieve their life targets in the host countries, failure to achieve their personal and professional goals may expose them to severe and prolonged psycho-physical stress^8^.

The feeling of being uprooted and culture shock are known drivers of mental health issues harms in migrant populations, with disorders that may be milder or more severe depending on several factors, including the voluntary or forced nature of the migration, the geographical distance traveled to migrate, and the reasons for migrating^9^. The most common mental disorders among migrants include schizophrenia^10–12^, anxiety and depression^13–15^, but most of all post-traumatic stress disorder (PTSD)^16,17^. Most of the migrants report experience of distress, sadness, fear, desolation, and hopelessness during and post displacement^18^.

Within migrant populations, particularly high rates of mental disorders have been registered among asylum-seekers and refugees^19,20^. In 2021, the number of refugees globally registered by the UNHCR continued to increase, reaching 21.7 million, against the 15 million of 2020^21^. In particular, in 2021, 66,770 people sought refuge in Italy, 97% more than in 2020^22^. The refugee experience is characterized by violence, war, and persecution, which can have long-lasting effects on their psychological well-being^21,23^. Migration and post-migration factors, which include life-threatening conditions during the travel and a limited social integration in the host country, may also affect the mental health conditions of asylum-seekers^24,25^. These observations support the thesis that the way in which refugees or asylum-seekers are cared after their arrival in the host country plays a relevant role in their mental health^26^.

Unfortunately, there is a paucity of studies on health trajectories among asylum-seekers and refugees during their stays at migrant reception centers. Particularly elusive are the effects on mental health, since the patients’ psychological state is rarely screened in a systematic way^27^, thus preventing an assessment of the quality of the assistance provided to migrants seeking asylum. To support health practitioner in their assistance in reception centers, another crucial advance would be the identification of possible socio-demographic factors and life experiences that may predict the insurgence of PTSD, which is particularly relevant among asylum-seekers, who may have been exposed to multiple traumatic events^28^. Understanding these factors could be critical towards the implementation of suitable public health policies for prevention and mitigation^29^.

Our study aims at relating mental health of asylum-seekers to both their past life experiences (before, during, and after the migration) and their first stay at a reception center. Specifically, the goal of this work is to address the following, fundamental research questions: (RQ1) what is the impact of the first contact with the reception system on asylum-seekers’ mental health? (RQ2) is it possible to identify risk (such as traumatic experiences) and shielding (such as family affections and education levels) factors for PTSD?

Our study contributes to answer these research questions by focusing on the first contact that asylum-seekers have with the healthcare systems of the host country. In particular, we examine the initial period that asylum-seekers spend in the reception facilities in Rome, Italy, under the coordination of the National Healthcare System. In these centers, migrants spent their 14-day COVID-19-related quarantine prescribed during the pandemic state of emergency, before being admitted to SAI (Sistema di Accoglienza e Integrazione, Italian for Reception and Integration System) network facilities. This short two-week stay represents the first contact with the national reception and integration system, thereby likely to bear a relevant impact on the mental health of migrants.

Through multiple questionnaires, we explore the mental health of 100 asylum-seekers hosted in these reception facilities. To answer RQ1, we test the hypothesis (H1) that the short, 14-day stay in the facilities had a positive impact on asylum-seekers’ mental health. Specifically, we studied the psychological well-being at the beginning and at the end of their period of stay in the facilities, in relation to a possible stress due to trauma. Confirming this hypothesis would provide further backing to and insight into the association between post-migration stressors in the settlement environment and psychological outcomes, such as depression, PTSD, and anxiety^30,31^.

We answer RQ2 by testing the hypotheses that: (H2-1) the lifetime experiences of asylum-seekers with PTSD were different than other asylum-seekers, and that (H2-2) past traumatic experiences and demographics of the asylum-seekers could help predict the insurgence of PTSD. To test these hypotheses, we administered migrants a demographic questionnaire, together with a set of four questionnaires (the Primary Care PTSD Screen for DSM-5^32^, the Harvard Trauma Questionnaire^33^, the Trauma and Loss Spectrum—Self Report^34^, and the List of Migration Experiences (LiMEs) questionnaire^35^) to detect the insurgence of (manifest or sub-threshold) PTSD and identify its possible roots.

We identified the traits that significantly differed in participant positive to PTSD by combining the results of the Harvard Trauma Questionnaire and of the Trauma and Loss Spectrum—Self Report. Then, we used supervised learning classification models to extract the possible predictors of its insurgence, thereby building a predictive model that pinpoints the risk and shielding factors of PTSD. This predictive model is used for a twofold purpose: (i) identifying the traumatic events that left the deepest marks on the psychological well-being of the asylum-seekers, and (ii) isolating the demographic factors, such as family affections and education levels, that might be associated with a better way of coping with migration related trauma.

The results of our study show that past trauma increases the likelihood of PTSD in migrants, and that their mental well-being improves when they stay in a secure and supportive place. This evidence highlights the need for appropriate policies to help migrants’ mental health when they reach host countries. Altogether, our findings represent a step towards informing public authorities in the design of effective medical support and shaping policy and practices in healthcare and migrants’ reception. These benefits could help channel the efforts of national and inter-governmental bodies towards the pressing challenge of mitigating the devastating effects of persecution and war on mental health^20^.

## Methods

**Fig. 1:**
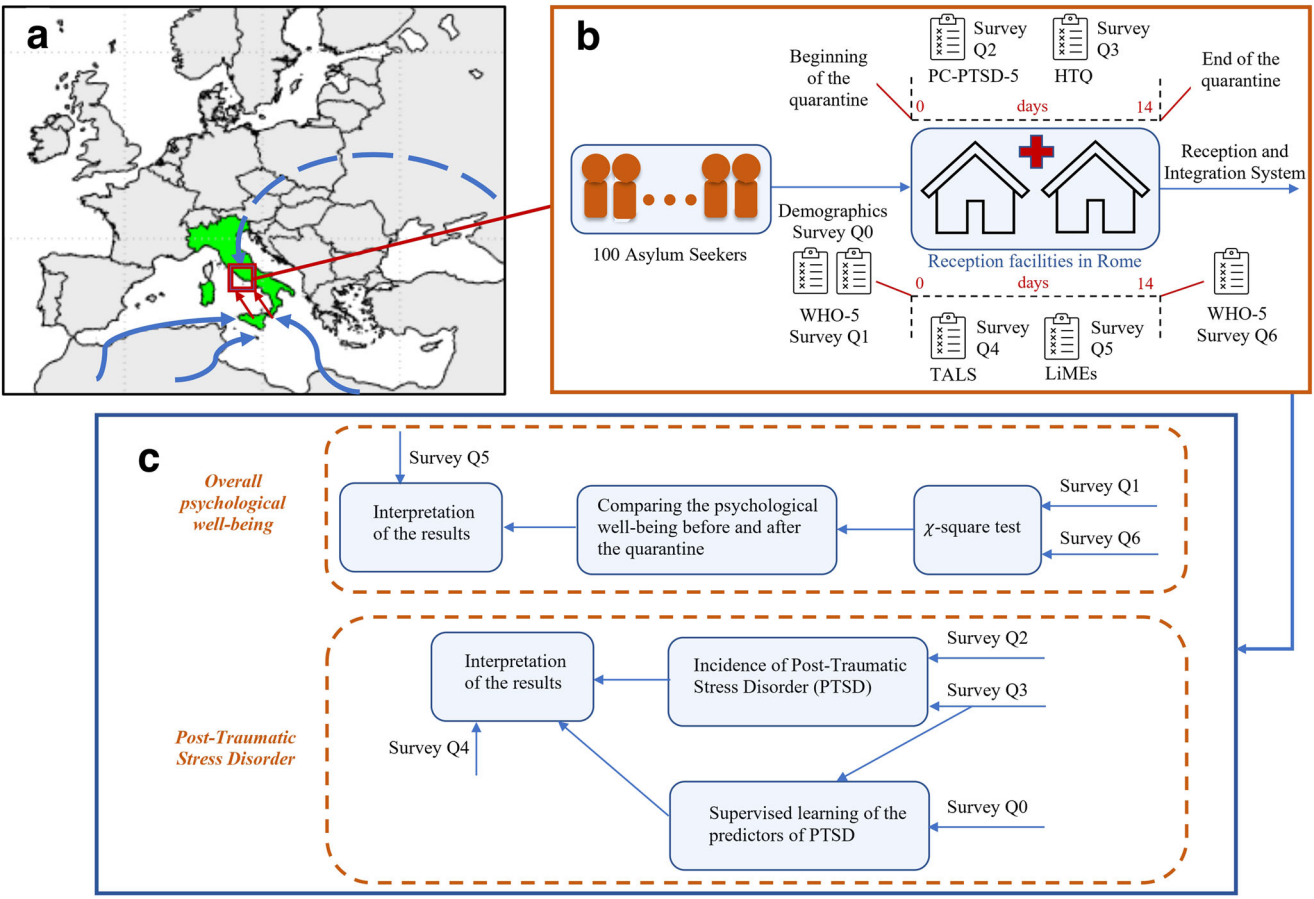
Schematic of the data collection and analysis. We focus on a sample of 100 asylum-seekers in Italy during their first contact with the national reception and integration system, which took place between May 2021 and September 2021 in Rome. In the schematic in (**a**), blue arrows correspond to migration flows to Italy, which is highlighted in green, whereas red arrows to internal transfers from hotspots to Rome, which is identified by a red square. As illustrated in (**b**), at their arrival in the reception facilities, the migrants were administered a demographic questionnaire (denoted Survey Q0) and the WHO-5 survey on psychological well-being (Survey Q1). During their 14-day COVID-19 related quarantine before the admission into the Reception and Integration System (in Italian, SAI—Sistema di Accoglienza Integrato), they were administered two surveys for detecting PTSD (namely, the PC-PTSD-5, Survey Q2, and the HTQ, Survey Q3), the Structured Clinical Interview for Trauma and Loss Spectrum (SCI-TALS, Survey Q4) to evaluate the spectrum of the stress response, and the Italian version of the LiMEs checklist of the traumatic experiences and living difficulties encountered before migrating, during the travel, or in the destination country (Survey Q5). At the end of their stay (day 14), the WHO-5 questionnaire (Survey Q6) was administered again to detect variation in the overall psychological well-being, as illustrated in (**c**). The incidence of PTSD were evaluated from Surveys Q2 and Q3, whereas supervised learning was used to identify the possible traumatic events (Survey Q3) or demographics traits (Survey Q4) that may help predict the insurgence of PTSD, see “Methods” for further details.

Here, we provide a brief description of the asylum system in Italy, of the target sample of participants, and of the questionnaires that have been administered to them. Then, we describe the supervised learning algorithms that were used to identify the best predictors of PTSD, and explain the metrics used to select the best classifiers. A schematic of the overall study is reported in Fig. 1.

### Asylum-seekers reception system in Italy

The asylum-seekers reception system is structured as follows. Initial aid and identification services to asylum-seekers is provided at the First Aid and Reception Centres (Italian acronym: CPSA), established in 2006, which now operate as hotspots. The government then provides assistance and reception to asylum-seekers either in existing collective governmental centers or in centers that are established by specific Ministerial Decrees. This includes centers previously known as governmental centers for accommodation of asylum seekers (Italian acronym: CARA) and accommodation centers (Italian acronym: CDA). Finally, a full and complete reception takes place in the System of Accommodation and Integration (SAI), which prioritizes vulnerable individuals and provides access as soon as possible. SAI programs offer various kinds of support for victims of trafficking, domestic violence, and exploitation; persons with medical needs; persons who have experienced natural disasters in their home country; persons who have performed acts of civic value; persons who have received special protection from the authorities; former unaccompanied minors who have obtained assistance from the authorities.

Our study is framed within this final step of the reception of asylum-seekers, where they can benefit from a range of services, including social and psychological assistance, cultural mediation, Italian language courses, legal information service, and information on territorial services. Beneficiaries of international and special protection also receive support for integration, job research, job orientation, and professional training.

### Participants

This study was conducted in the reception facilities for asylum-seekers based in Rome (coordinated by the Municipality of Rome, supported, regarding medical assessments, by Migrant Health Unit of the Local Health Authority Rome 2 - National Healthcare System) from May 2021 to September 2021. Migrants were required to quarantine for 14 days to comply with the regulations in effect during the COVID-19 pandemic. During the time-span of the study, 150 asylum-seekers were admitted in the facilities, and during the preliminary medical checks were asked if they agreed to the administration of the questionnaires for checking their psychological well-being, and to participate in the study.

Out of the 150 asylum-seekers, 100 agreed to participate and completed all the questionnaires, which were administered by a psychiatrist or by a psychologist supported by a cultural mediator, who helped accounting for cultural issues in the interpretation of Western questionnaires and diagnostic systems. If from the interview or from the outcome of the questionnaire the clinical staff detected possible vulnerabilities, further interviews were scheduled with psychiatrists and psychologists of the Center for Mental Health Care in Rome for a further evaluation and, if needed, for an all-round assistance by the National Health Care System. During the study, a psychologist and a psychiatrist were always available for possible emergencies, although migrants have never needed urgent support.

Next, we detail the procedure followed to secure consent for the participants. Before starting the study, we obtained informed consent from each participant. A cultural mediator who spoke the same language as the participant explained the study and the written Consent Form to them. The participant signed the Consent Form only if they agreed to take part in the study. We also told them that they had the right to choose whether or not to participate in the study, and that their decision would not affect their situation in any way. We assured them that they could change their mind and stop participating at any time, without any consequences.

Note that, albeit the asylum-seekers were at their first contact with the national reception and integration system, they were not newly arrived, and the 94% of them had lived in Italy for at least 1 year. Indeed, access to the reception and integration system is not always simultaneous with arrival in Italy. Many asylum-seekers, once they arrive in the host country, may live in a condition of legal invisibility before completing all the legal steps to have their political refugee status recognized. This time can also be long, and, especially during the pandemic period, international protection requests have suffered delays.

The current research was conducted in accordance with the Declaration of Helsinki. The local ethical review board (Comitato etico Lazio 2, ASL Roma 2) approved the study (identified with number 166.20) with protocol 0029199. All data analyzed in the study are publicly available at ref. ^36^.

### Cultural mediators

The interaction between mental health professionals and asylum-seekers is often hindered by language and cultural barriers, which increase the risk of misunderstanding and misdiagnosis. This is even more evident when asylum-seekers have experienced trauma, where peculiar expressions to explain distress, anguish, or anxiety may be inaccurately interpreted as resistances to communication or even as psychotic symptoms. Culturally contextualized understanding of trauma has a pivotal role when working with refugee populations, and cultural interpretation and mediation are necessary in the healing process^37^. Cultural mediators are not mere interpreters, as they often become co-therapists working in synergy with the other health professionals. Their role extends far beyond that of translation to include negotiations, facilitations, and empathetic emotional listening to help displaced populations build adaptive strategies to cope with their situation^38^.

In this study, cultural mediators were full-time employees of the project recruited based on their language skills and work experience through non-profit organizations. All of them had a translation or cultural mediation certificate or diploma. The cultural mediators who took part in the research were specifically trained and qualified health workers, who were working at the Local Health Authority, where the study was conducted, for several years. They constituted a crucial professional figure, embodying the fundamental link between the therapist/physician and the patient. We assigned to each migrant a cultural mediator from the same geographical and cultural area to promote cultural relevance and understanding.

The specific competences of cultural mediators recruited for this study ensured a safe environment, respectful dialogs, and a collaborative workplace. In their interactions with migrants, cultural mediators were aware of the differences in cultural contexts between the country of origin of the migrants and their host country^39^. In particular, they were trained to consider the cultural specificity of the stigma towards mental disorders, the sanctity of confidentiality, and the need to convey information and formulate questions in a non-technical, yet clear, language. To ensure continuity of support, each migrant was followed by the same cultural mediator in the 14-day period (and beyond the duration of this study).

### Questionnaires

Participants (Female: 6%, age 30 ± 7.2 years) were asked to answer six different surveys during their two-week stay in the facilities, and the full tables with the anonymized replies to each question of the surveys are reported in ref. ^36^. In order to address the two research questions of this study, we utilized surveys that could provide a complete picture of the demographics of the participants, detect the insurgence of PTSD, and identify possible root causes of PTSD in past and present traumatic experiences and living conditions of the participants. More specifically, the select questionnaires were:

Survey Q0: a demographic survey, administered to collect the main demographic information on the participants, including, among others, sex, age, marital status, education, wealth, and ties with the society of origin and destination. The salient outcomes of the survey are reported in Tables 1–3, whereas complete results can be found in ref. ^36^.

**Table 1:** Select demographic traits of the participants (part 1).

Employment status		Employed	Unemployed	
In the origin country		90%	10%	
In Italy		36%	64%	
Ease in getting through the month		Very/quite hard	Very/quite easily	
In the origin country		73%	27%	
In Italy		80%	20%	
Support from relatives		Yes	No	
In the origin country		52%	48%	
In Italy		97%	3%	
Support from institutions				
In the origin country		50%	50%	
In Italy		94%	6%	
Supportive partner/friends		None	Few	Many
In the origin country		79%	21%	0%
In Italy		69%	19%	12%
Supportive acquaintances				
In the origin country		71%	24%	5%
In Italy		68%	28%	4%
Social activism		No	Little	Constant
In the origin country		65%	19%	16%
In Italy		74%	17%	9%
Engagement in sports/hobbies	Never	Rarely	Often	All the time
In the origin country	16%	19%	44%	21%
In Italy	31%	31%	32%	6%

Employment status, ease in getting through the month, supportive relatives, supportive partner/friends, supportive acquaintances, support from institutions, social activism, and engagement in sports/hobbies in the periods lived in the origin country and in Italy.

**Table 2:** Select demographic traits of the participants (part 2).

Country of origin	Nigerian 24%	Somalian 8%	Gambian 7%	Pakistan 7%	Other 54%
Civil status		Unmarried 68%	Married 29%	Separated 3%	
Children		None 65%	Cohabiting 28%	Non-cohabiting 7%	
Education level	None 14%	Primary school 19%	Junior high school 46%	High school 15%	Higher 6%
Country of education		Italy 6%	Other 79%	No education 15%	
Years lived in Italy (years)		years <1: 6%	1 ≤ years ≤ 5: 80%	years >5: 14%	

Country of origin, civil status, children, level of education, country of education, and total number of years lived in Italy.

**Table 3:** Select demographic traits of the participants (part 3).

Attendance at	Never	Sometimes	Often
Ethnic events	35%	50%	15%
Inter-ethnic events	40%	51%	9%
Meetings of migrants’ associations	69%	29%	2%
Public demonstrations	79%	18%	3%

Frequency of attendance at ethnic or inter-ethnic events, meetings of migrants’ associations, and public demonstrations since their arrival in Italy.

Survey Q1, Q6: WHO-5 well-being index, administered both at the beginning and at the end of the reception period. The scope of this questionnaire is to assess subjective psychological well-being, and since its publication in 1998, it has been the most widely used and has been translated in more than 30 languages^40^. We referred to it as Q1 when referred to answers collected on day 1, and as Q6 when referred to answers collected at the end of the stay, that is, at day 14.

Survey Q2: the Primary Care PTSD Screen for Diagnostic and Statistical Manual of Mental Disorders 5 (DSM-5), often shortened as PC-PTSD-5^32^ is a five-item screen designed to detect individuals with probable PTSD in primary care or in other medical settings. The first item assesses lifetime exposure to traumatic events, and the score is 0 if the respondent denies exposure. Otherwise, the respondent is administered five additional binary questions about how the trauma exposure affected them during the past month. For primary care use, those screening positive should then be assessed with a structured interview for PTSD. In our study, all participants were further tested for PTSD.

Survey Q3: HTQ—Harvard Trauma Questionnaire; it was constructed by Mollica et al. as a bilingual test of PTSD^33^, modeled after the Hophs Symptom Checklist-25^41^. The questionnaire, although originally targeted at southern Asian populations, was later used for diverse refugee groups^42–44^. It is composed of a range of questions on traumatic events, DSM-related post-traumatic symptoms, and additional, culture-related post-traumatic symptoms.

Survey Q4: TALS—Trauma and Loss Spectrum—Self Report. This survey investigates soft signs, low-grade symptoms, subthreshold syndromes, and temperamental and personality traits that comprise clinical and subsyndromal manifestations of PTSD. It includes 116 questions exploring the lifetime experience of a range of loss and/or traumatic events and lifetime symptoms, behaviors and personal characteristics that might be manifestations or risk factors for developing a stress response syndrome. The questionnaire is organized into 9 domains, whose detailed description is reported in ref. ^34^. Each question has a binary (yes/no) answer, and the scores in each domain are obtained by counting the number of positive answers.

Survey Q5: LiMEs—Italian version^45^. It is a checklist of 59 events that the participants to the study might have experienced before migrating, during the travel, or after the arrival in the destination country. The items of the list are organized in two groups: traumatic experiences (that is, war/conflicts, intentional traumas, witnessing of traumatic events) and living difficulties (that is, difficult access to assistance, unemployment, poverty, and legal issues).

### Statistics and reproducibility

To answer RQ1 and study whether the 14-day stay at the facilities had a positive impact on asylum-seekers (H1), we compared the subjective psychological well-being of the asylum-seekers at the beginning and at the end of their stay in the reception center by performing a McNemar exact test^46^ for matched observations. The null hypothesis of the test is that the probability of individuals being positive to Q1 is the same as that of being positive to Q6. We used a significance level *p* = 0.05. We note that a sample size of 30 would be needed to obtain a power of 0.8 for this test^47^. In our case, we have *N* = 100 participants, so that the power of the test is larger than 0.999.

To answer RQ2, we identified possible traits that differentiate participants positive for PTSD according to Q3, by comparing their scores in the various domain of Q4 against the scores of the participants that tested negative for PTSD (H2-1). Towards this aim, we performed the Mann–Whitney *U*-test^48^ with a significance level *p* = 0.05. For a statistical power 0.8, we would need a sample size of 29^49^, which is smaller than the number *N* = 100 of participants to the study. Hypothesis H2-2 was instead examined by using supervised learning, as explained in the following.

### Supervised learning algorithms to predict PTSD

We used supervised learning algorithms to extract the features that allow us to best predict the insurgence of PTSD, thereby testing H2-2. More specifically, we considered the answers provided to the questions related to demographics (questionnaire Q0) and to traumatic events experienced in the country of origin (Section C of Q3) as possible predictors of PTSD (Section D of Q3). Then, we used classifiers from supervised learning to extract the salient features that allowed us to predict whether an individual was subject to the PTSD. The pipeline for data analysis^50,51^ follows the four steps detailed below.

Step 1: data preprocessing. The survey data were manually entered by the practitioners who administered the survey, whereby the datasets were subject to unavoidable inconsistencies. Therefore, before applying machine learning algorithms, we cleaned the dataset by removing duplicates and resolving the inconsistencies (for example, we homogenized capital/lower letters cases, and corrected wrong inputs in the birth-date format). Data cleaning was then followed by data transformation, through the application of generalization. For example, we converted the granular data in the job field to high-level information by using concept hierarchies, that is unemployed/jobless → jobless whereas cook/hairdresser/mechanic → employed. In addition, when needed for some analyses, we aggregated data (for example, for the question “How long have you been in Italy?,” the answers “less than one year” and “1–5 years” have been merged into “less than 5 years” whereas “6–10 years, 11–15 years” and “more than 15 years” into “more than 5 years”).

Step 2: feature selection. In classification algorithms, it is important to select a subset of relevant attributes to avoid considering an excessive number of features, which could lead to overfitting problems. Namely, only attributes that significantly enhance the predictive power of our classifier should be retained. Here, we employ a univariate feature selection method based on *χ*^2^-tests^52^. Specifically, we perform a *χ*^2^-test of independence between each of the features and the response variable, and rank the features according to $-\log (p)$, with *p* being the *p* value of each test, and $\log$ being the logarithm base 10. Indeed, a small *p* value indicates that the corresponding predictor variable is dependent on the response variable, and therefore is an important feature. In our analysis, a feature is selected if the *χ*^2^-test of independence yields a *p* value of at most 0.05, see Supplementary Table S1.

We also tested the Minimum Redundancy Maximum Relevance (MRMR) and ReliefF algorithms as alternative feature selection algorithms^53,54^. However, the MRMR algorithm yielded worse predictive power for our classifier (see Supplementary Notes 1), whereas the ReliefF algorithm was too sensitive to the selection of its key parameter, that is, the number of neighbors of each observation, see Supplementary Table S2.

Step 3: classification models. Supervised learning algorithms build a mathematical model of a set of data that contains both the predictors and the response variable. We considered as response variables the symptoms of the PTSD disorders, identified through the questions of the PC-PTSD and HTQ Questionnaires (surveys Q2 and Q3) reported in Tables 4 and 5, and as possible predictors the traumatic life events identified through Section C of Survey Q3 (see Table 6) and the answers to questions 2–5, 7–10, 12, and 15–30 of the demographic Survey Q0. The response variables have been binarized, by assigning “0” to the answers “Not at all” and “A little,” and “1” to the answers “Quite a bit” and “Extremely.”

**Table 4:** Answers and outcome of the Primary Care PTSD Screen for DSM-5 (survey Q2).

Item	Yes
Have had nightmares about it or thought about it when you did not want to?	35%
Tried hard not to think about it or went out of your way to avoid situations that reminded you of it?	36%
Were constantly on guard, watchful, or easily startled?	27%
Felt numb or detached from others, activities, or your surroundings?	29%
*Positive to PTSD*	22%

Participants are considered positive to PTSD if they answer “yes” to at least three of the four questions.

**Table 5:** Distribution of the answers to Section D of Harvard Trauma Questionnaire (survey Q3).

Symptoms	Not at all	A little	Quite a bit	Extremely
Recurrent thoughts or memories of the most hurtful or terrifying events	32%	26%	25%	17%
Feeling as though the events happening again	53%	18%	21%	8%
Recurrent nightmares	48%	24%	16%	12%
Feeling detached or withdrawn from people	58%	15%	16%	11%
Unable to feel emotions	65%	20%	7%	8%
Feeling jumpy, easily startled	49%	27%	14%	10%
Difficulty concentrating	47%	21%	14%	18%
Trouble sleeping	43%	24%	10%	23%
Feeling on guard	58%	13%	21%	8%
Feeling irritable or having outbursts of anger	56%	21%	15%	8%
Avoiding activities that remind you of the traumatic or hurtful event	55%	16%	15%	14%
Inability to remember parts of the most hurtful or traumatic events	61%	23%	10%	6%
Less interest in daily activities	65%	13%	10%	12%
Feeling as if you don’t have a future	66%	8%	12%	14%
Avoiding thoughts or feelings associated with the traumatic or hurtful events	50%	20%	16%	14%
Sudden emotional or physical reaction when reminded of the most hurtful or traumatic events	52%	20%	14%	14%
*Positive to PTSD*	23%			

The answers are scored as follows: 1 for “not at all,” 2 for “a little,” 3 for “quite a bit,” and 4 for “extremely.” The score for each participant is given by the sum of the scores of each answer. Participants are considered positive if their score is larger than 40.

**Table 6:** Answers of Section C of Harvard Trauma Questionnaire (survey Q3).

Trauma	Incidence
Material deprivation	70%
War-like conditions	69%
Bodily injury	50%
Forced confinement and coercion	50%
Torture	52%
Forced to harm others	12%
Disappearance, death, or injury of loved ones	51%
Witnessed violence to others	61%
Brain injury	8%

The table reports the incidence of the listed traumas among the participants.

Since the response variables take value in a finite set, we used a particular class of supervised learning models, the classification algorithms. Specifically, we trained and validated algorithms belonging to the following families^50,51^: (i) Support-Vector Machines (SVM) algorithms, which extract models that maximize the width of the gap between categories in the data, to minimize the generalization error of the classifier; (ii) Decision tree algorithms, commonly used in data mining, which sort the features in a tree structure, where the leaves correspond to the possible outcomes of the prediction; a key element in these algorithms is the choice of the hierarchy among the features, and a greedy divide and conquer strategy is employed to identify the optimal split points within the tree; (iii) the family of ensemble learning algorithms, which include all the methods that combine multiple learners to make a decision, typically in supervised machine learning tasks; the premise of ensemble learning is that the combination of multiple models is likely to reduce the errors of a single learner, thereby increasing the overall prediction performance^55^; and (iv) *k*-Nearest Neighbor (*k*-NN) algorithms, which classify an object by assigning it to the class most common among its *k* closest training examples in a data set; in particular, they assign weights to the contributions of the neighbors, so that closer neighbors contribute more to the average than the more distant ones^56^.

For each of these four families, we considered different algorithms (see Supplementary Table S3 for a full list), and also a modified cost function to compensate for the imbalanced sample, where we associated to each class of classification a weight inversely proportional to the class frequency in the input data. To prevent overfitting, for each algorithm we followed a 10-fold cross-validation scheme, which consists in dividing the sample into a training and validation set of 90 and 10 elements, respectively. We repeated the procedure 20 times and computed the average performance of each algorithm, according to the metrics listed in the following step 4.

Step 4: performance metrics and algorithm selection. Here, we recall that a true positive corresponds to a case in which the algorithm correctly identifies a positive. For instance, we have a true positive when the algorithm predicts that a participant has PTSD and this is actually the case. Similar definitions hold for true negative, false positive, and false negative.

We evaluate the performance of each algorithm according to the following, standard metrics: sensitivity, or true positive rate, is the ratio between the number of true positives (TP) and the total number of positives (P); fall-out, or false positive rate, is the ratio between the false negatives (FN) and the total number of negatives (N); specificity, or true negative rate, is the ratio between the true negatives (TN) and the total number of negatives N; accuracy is the ratio between the correct classifications and the total number of samples, computed as (TP+TN)/(P+N); Area Under the Curve (AUC) is the area under the Receiver Operating Characteristic (ROC) curve, obtained by plotting sensitivity versus specificity at different values of the classification threshold. A random classifier would yield AUC = 0.5.

Among all the classification algorithms, we chose those maximizing the sensitivity, given that the accuracy was always larger than 70%, the AUC larger than 0.70, and the fall-out lower than 0.25. The choice of privileging sensitivity is for prioritizing the detection of as many subjects with PTSD as possible.

### Reporting summary

Further information on research design is available in the Nature Portfolio Reporting Summary linked to this article.

## Results

In what follows, we report the answers to the two research questions formulated in this study.

### Answer to RQ1: sheltering in refugee centers has a positive impact on migrants’ mental health

The participants of this study spent two weeks in the reception facilities. To test whether the permanence in a center improved their psychological well-being, the WHO-5 survey was administered twice, once at the arrival of the migrant (survey Q1) and once 14 days later, when they left the facility (survey Q6). The results of the surveys administered at the arrival of the migrants show that 51% of the participants needed to undergo further psychological tests. This percentage dropped to 21% at the end of their stay at the refugee center, suggesting that living in a shelter where people took care of their physical and mental needs had a positive impact on mental health: a McNemar exact test rejected the null hypothesis that the probability of being positive to Q1 and Q6 are the same with *p* < 0.001.

To further delve into this result, we looked into the answers to each item of the survey. Namely, each answer referred to time spent experiencing a given (positive) feeling. We binarized the answers, and associated “1” to “positive answers” corresponding to “more than half of the time/most of the time/all the time,” whereas “0” to “at no time/some of the time/less than half of the time.” As evident from Table 7, there is a general improvement in the reply to each question, with the exception of question 3, where the fraction of positive answers was almost unchanged (for the full, non-dichotomized distribution of the answers we refer the reader to ref. ^36^). A further indicator that the stay in the refugee centers improved mental health is that, out of the eight participants who tested positive to Q1 and also to the PTSD tests Q2 and Q3, five showed an improved psychological well-being, whereby the results to the WHO-5 test administered at the end of their stay (Q6) was negative.

**Table 7:** Distribution of the answers to the questions of the WHO-5 survey administered at the arrival of the migrant (Survey Q1) and when they left the facility 14 days later (Survey Q6).

Item	Less than half of the time	More than half of the time
I felt cheerful and in good spirit	30% [15%]	70% [85%]
I felt calm and relaxed	24% [13%]	76% [87%]
I felt calm and vigorous	17% [18%]	83% [82%]
I woke up feeling fresh and rested	28% [17%]	72% [83%]
My daily life is filled with things that interest me	36% [26%]	64% [74%]

Percentages between square brackets correspond to the answers provided at the exit from the facility.

Overall, the outcome of the WHO-5 survey confirms hypothesis H1 that the 14-day stay in the facilities positively impacted the asylum-seekers’ mental health. It is interesting to point out that this increased psychological well-being was achieved albeit the employment level and the connections with the family of origin reduced when they arrived in Italy, see the demographic traits in Table 1 and the questions extracted by the LiMEs questionnaire (survey Q5) and reported in Table 8. For instance, the fraction of unemployed and homeless individual increased, and the distance from their family members dramatically increased the feeling of loneliness. These factors represent important stress factors which, when combined with pre-existing factors, can lead to physical or mental disorders^57^. This additional socioeconomic burden appears to be compensated by the greatest support and assistance that they claim to have received since their arrival in Italy, see again Tables 1 and 8.

**Table 8:** Answers of LiMEs Questionnaire (survey Q5).

Item	Before	During	After
Food and water shortages	27%	55%	3%
Impossibility to get the food you like	22%	51%	11%
Not having your own home	11%	34%	45%
Having no shelter	10%	58%	14%
Poverty	29%	30%	31%
Little help from the government	44%	37%	9%
Little help from charities	31%	30%	12%
Overcrowding of accommodation places	5%	39%	41%
Impossibility to find a job	5%	15%	59%
Unemployment	6%	10%	77%
Poor working conditions	5%	27%	48%
Language difficulties	0%	11%	64%
Loneliness and boredom	4%	15%	51%
Feeling deprived of something	15%	25%	39%
Feeling of experiencing inequities	34%	34%	12%
Concern about the family left at home	3%	14%	61%
Impossibility to go back home in emergencies	2%	18%	51%

List of traumas experienced by at least 70% of the participants. Each column specifies which percentage of the participants experienced each trauma before the migration, during the travel, or after the arrival in Italy, respectively.

These results highlight the importance of understanding the socio-psychological values and cultural narratives of migrants, enhancing their resilience, and giving them the possibility to experience positive psychological change, appreciate a new experience of calm and relax, good relationships with others, new possibilities in life, personal strength, and spiritual change (see Table 7), in line with previous studies^58^.

### Answer to RQ2

A first step towards answering RQ2 was to assess the incidence of PTSD among the migrants hosted in the center, which we evaluated by administering the Harvard Traumatic Questionnaire HTQ, labeled as survey Q3. The distribution of the answers to each question of the tests is reported in Table 4. The questionnaire showed a 23% incidence of the PTSD, which is much higher than that in the general population, ranging between 4% and 10%^59,60^. Since individual with high lifetime exposure to trauma have a 35% incidence of PTSD^60^, a possible explanation of the incidence observed in our sample could be that some of the participants were exposed to traumatic experiences. Note that the results of the PC-PTSD-5 test (questionnaire Q2) used for primary care screen of PTSD is in agreement with the results of the HTQ in the 83% of the cases, and output a similar incidence of 22% (the distribution of the answers to each question of the test is reported in Table 5). Next, we tested hypothesis H2-1 to determine whether the lifetime experiences of asylum-seekers with or without PTSD were different.

### Asylum-seekers with PTSD reported more traumatic events and personality characteristics related to loss and trauma

To examine H2-1, we compared the results of the HTQ against the results of the Structured Clinical Interview for Trauma and Loss Spectrum (SCI-TALS) developed to evaluate the spectrum of the stress response, labeled as Q4 in this study (see the Methods for a description of the survey and^36^ for the participants’ answers). Interestingly, we found that, in all the domains of Q4, the median of the scores of individuals who tested positive to PTSD according to the HTQ was higher than that of those who tested negative. Specifically, for each domain we performed a left-tailed Mann-Whitney U-test, with *n*_1_ = 23 and *n*_2_ = 77 being the number of individuals who tested positive and negative to the HTQ test for PTSD, respectively, and obtained the following results: domain I, median 0.5 vs 0.3 (*U* = 356.5, *p* < 0.001); domain II, median 0.59 vs 0.22 (*U* = 376.5, *p* < 0.001); domain III, median 0.48 vs 0.29 (*U* = 462, *p* < 0.001); domain IV, median 0.72 vs 0.28 (*U* = 307, *p* < 0.001); domain V, median 0.78 vs 0.22 (*U* = 298, *p* < 0.001); domain VI, median 0.58 vs 0.25 (*U* = 367.5, *p* < 0.001); domain VII, median 0.25 vs 0 (*U* = 480, *p* < 0.001); domain VIII, median 1 vs 0 (*U* = 211, *p* < 0.001); and domain IX, median 0.17 vs 0 (*U* = 555, *p* = 0.002).

These results confirm the clinical relevance of partial or subthreshold forms of PTSD in migrants. Individuals who tested positive to PTSD reported more loss events, grief reactions, traumatic events, avoidance symptoms, numbing, arousal, maladaptive coping and personality characteristics that might be related to loss and/or trauma, thereby confirming hypothesis H2-1. From a clinical point of view, it is important to precisely assess any trauma or loss as symptoms might be mistakenly confused with those of depressive disorders, especially in the case of subthreshold PTSD.

### Past traumatic events, not demographics, serve as predictors of PTSD in asylum-seekers

A relevant fraction of the participants in the study were subject to traumatic life events that may have been the trigger of the observed post traumatic stress disorder, as illustrated by their replies to Section C of Survey Q3, see Table 6. At the same time, one could argue that the presence of family ties or a high degree of integration in the society may shield them from the consequences of their trauma. Here, we use the classification models introduced in the Methods to test hypothesis H2-2 by identifying the best predictors of PTSD and revealing the possible presence of shielding demographic factors. In particular, we considered as possible predictors the answer to Section C of Survey Q3, and answers 2–5, 7–10, 12, and 15–30 of Survey Q0. As response variables, we considered, one-by-one, the responses to each of the questions reported in Tables 4 and 5, which are the questions that investigate the symptoms of PTSD, and were used to detect its presence using the PC-PTSD and HTQ tests, respectively.

As for the PC-PTSD questions, we observed that none of the classifiers we built is capable of predicting the answers with a level of accuracy, sensitivity, fall-out and AUC (Area Under the Curve) compatible with the criteria we indicated in the sub-section “Performance metrics and algorithm selection” of the Methods section, thereby implying that neither the traumatic events nor the demographics have a sufficient predictive power. Similarly, when trying to predict the answers to the HTQ test for PTSD, we noticed that demographic variables did not help the prediction. Indeed, they were never extracted as salient features by any of the classification algorithms, and they did not enhance the prediction accuracy.

The traumatic events were instead effective in predicting the presence of recurrent thoughts, the feeling of being easily startled, and sudden reaction to traumatic memories, see Table 9. In particular, a quadratic SVM predictor selected the events “war-like conditions,” “torture,” “forced to harm others,” “disappearance, death of injury of loved ones,” and “witnessed violence to others” as key predictors of recurrent thoughts (accuracy 0.85, sensitivity 0.86, fall-out 0.16, AUC = 0.84). All the traumatic events were used by a RUS-Boosted Trees model (accuracy 0.80, sensitivity 0.82, fall-out 0.19, AUC = 0.84) and by a Medium Tree model with modified cost function (accuracy 0.76, sensitivity 0.72, fall-out 0.21, AUC = 0.75) to predict the feeling of being easily startled, and the sudden reaction to traumatic memories, respectively.

**Table 9:** Extracted classification models to predict Post Traumatic Stress Disorder from life events.

Traumatic symptom	Model	Accuracy	TPR/FPR	Traumas	AUC
Recurrent thoughts or memories of the most hurtful or terrifying events	SVM (Quadratic)	0.85	0.86/0.16	2.5-8	0.84
Feeling jumpy, easily startled	Ensemble (RUSBoosted Trees)	0.80	0.82/0.19	All	0.84
Sudden emotional or physical reaction when reminded of the most hurtful or traumatic events	Tree (Medium Tree) Modified cost function	0.76	0.72/0.21	All	0.75

None of the other symptoms were successfully predicted by the traumatic life events, whereby the accuracy, sensitivity, fall-out, and AUC did not match our selection criteria. This might be due to either the relatively small sample of participants, or to other causes of these symptoms that have not been captured by any of the administered questionnaires. Finally, we remark that, as explained in the “Methods,” we have binarized the response variables, since none of the predictors would have met the selection criteria, otherwise.

## Discussion

The migration process is potentially disruptive to the mental balance of an individual. This is even more true for forced migrations, where individuals have to abandon their homes due to natural disasters, wars, or persecutions, and the trauma associated to the migration process adds up to those experienced in their place of origin. In this context, when the refugees and asylum-seekers arrive in the host country, it is crucial to take care not only of their physical wounds, but also of their mental health. In fact, asylum-seekers are characterized by a much higher incidence of several mental disorders, and in particular of PTSD.

Machine learning techniques are gaining momentum as detection tools for mental health problems^61^. Not only do they help understand psychiatric disorders, but also they can distinguish and classify mental health problems among patients for further treatment^62^. However, little is known about the factors that may predict the insurgence of mental disorders in migrant populations. Preliminary studies investigated how environmental conditions in the host country affect the psychological well-being of migrants^63^, but the impact of the first support received in the host countries, and of the experiences lived before, during, and after the migration had not been clarified yet. In this study, we have investigated the importance of an immediate support for the asylum-seekers by monitoring their mental health during their stay in a reception center in Italy, and used a supervised learning approach to seek for possible factors in their life experience that may be predictors of the insurgence of PTSD.

In accordance with previous study^64^, our research reveals that pre-migration factors (that is, traumas experienced in the places of origin) are crucial predictors of PTSD, but also point out that the reception in suitable structures where their mental health is monitored (as opposed to precarious reception conditions in large, isolated facilities) may mitigate the subjective psychological well-being of migrants. Indeed, our predictive model, based on supervised learning, does not point to the presence of any key demographic shielding factor. For instance, social ties or education level are never selected by our classifiers as salient features to predict the insurgence of PTSD, in contrast to previous traumatic experiences that are systematically selected. At the same time, we observe that the subjective psychological well-being of migrants is significantly improved at the end of their stay at the reception facilities, where they are welcomed by a reassuring environment and their physical and mental needs are monitored.

These results are in agreement with the fact that migrants who were forced to leave their homes are likely to experience stressors which may lead to mental health problems, underlining that pre-migration exposure to trauma is an important risk factor for developing mental health disorders. Crucially, our quantitative analysis also supports the thesis that post-migration factors play a crucial role in the adaptation and recovery from pre-migration trauma^18^. Primary care services and multidisciplinary teams should be tailored to meet the initial healthcare needs of newly arrived migrants^65^. Symptom reduction and improvement of functioning, as well as attention to the patients’ diverse health concerns and social and cultural needs, should be a continued focus for mental health providers^66^. Prompt psychological interventions to improve mental health, quality of life, functioning, and adaptation to the host country are of pivotal importance. In agreement with the existing theories^18,65^, our results provide quantitative backing to the importance of first reception, supporting the thesis that the mental healthcare needs of migrants should be assessed soon after resettlement, and adequate care should be provided, using a trauma informed approach, showing kindness and empathy during all encounters and taking a holistic, person-centered approach.

The present study also shows that bodily injury, torture, and witnessed violence to others are features that constantly appear as good predictors for PTSD symptoms, thereby urging for specifically tailored programs for migrants aimed to help participants develop social connections, self-esteem, self-efficacy, personal health, and safety skills. In particular, failure to address the post-migration stressors might undermine the recovery process from traumatic experiences and might limit the effectiveness of mental health care in refugee settings. Resources are needed to prevent trauma-related mental disorders in asylum-seekers and refugees, enhance mental health and well-being outcomes, and facilitate independence of these groups.

Overall, the results of this study point to the relevance of the immediate post-migration environment, suggesting that inadequate reception conditions in large facilities may have detrimental effects on asylum-seekers’ mental health. We recommend for host countries to implement reception models that provide effective protection and integration to this vulnerable population^67^. This work presents a first quantitative backing to a well-developed body of literature of international standards, guidelines, and best practices for refugee settings^68^. Future work should be targeted at confirming the results of this pilot study on different migrant samples. Given the observed need for a suitable post-migration reception, the importance of a timely support to migrants should also be explored. Existing delays in the access to the reception and integration system presently cause a vast majority of the asylum-seekers to live in a condition of legal invisibility for more than one year, before receiving an all-round assistance. Comparative studies could help choose between alternative paradigms for mental health monitoring and support of incoming migrants.

The findings of this study call for further research over a longer time horizon on the very approaches to diagnose mental health of migrants and identify ways towards assisting and supporting them. It is tenable that the use of cultural mediators was one of the reasons for the improved mental health of migrants in the 14-day stay. Longer studies may focus on the specific role of personalized, culturally contextualized interactions with migrants on their overall mental health, in line with studies in cross-cultural psychology^69,70^. Albeit the use of cultural mediators mitigated some of the drawbacks associated with the use of western diagnostic tools^71^, further efforts in this direction should be pursued. Within this context, we envision citizen science initiatives that would involve migrants in the design of the questionnaires to best capture their needs and offer a more comprehensive approach to appraise their mental health.

### Supplementary information

Reporting Summary

Supplementary Information

## Data Availability

The survey data that support the findings of this study are publicly available at ref. ^36^.

## Code availability

Code used to process the data is available at https://osf.io/9geqc/^72^.
