# Supplementary material for: Migrants’ mental health recovery in Italian reception facilities
Source: Commun Med (Lond). 2023 Nov 22;3:162. doi: 10.1038/s43856-023-00385-8 (PMC10665420; doi:10.1038/s43856-023-00385-8)
Supplement: Supplementary file 2 — Supplementary Information [file 43856_2023_385_MOESM2_ESM.pdf]

Supplementary Information

accompanying the paper

Migrants' mental health recovery in Italian reception facilities

Emanuele Caroppo[1*†], Carmela Calabrese[2,3†], Marianna Mazza[4,5], Alessandro Rinaldi[6], Daniele Coluzzi[6], Pierangela Napoli[6], Martina Sapienza[7], UOC Salute Mentale[‡], Maurizio Porfiri[8*], Pietro De Lellis[2*]

1 Department of Mental Health, Local Health Authority Roma 2, Rome, Italy
2 Department of Electrical Engineering and Information Technology, University of Naples Federico II, Naples, Italy
3 Institut de Neurosciences des Systèmes (INS), Aix Marseille Université, UMR1106, Marseille, France
4 Institute of Psychiatry and Psychology, Department of Geriatrics, Neuroscience and Orthopedics, Fondazione Policlinico Universitario A. Gemelli IRCCS, Università Cattolica del Sacro Cuore, Rome, Italy
5 Department of Psychiatry, Università Cattolica del Sacro Cuore, Rome, Italy
6 Migrant Health Unit, Local Health Authority Roma 2, Rome, Italy
7 Department of Life Sciences and Public Health, Università Cattolica del Sacro Cuore, Rome, Italy
8 Center for Urban Science and Progress, Department of Mechanical and Aerospace Engineering, and Department of Biomedical Engineering, New York University Tandon School of Engineering, Brooklyn, NY, USA

†These authors also contributed equally to this work.
‡A list of authors and their affiliations appears at the end of the paper
* emanuele.caroppo@aslroma2.it, mporfiri@nyu.edu, pietro.delellis@unina.it

Supplementary Notes 1. Alternative methods for feature selection.

As explained in the main text, a feature is selected as a potential predictor of PTSD only if the χ^2-test of independence yields a p-value of at most 0.05. Here, we report in Table 1 the list of features of survey Q3, section C, that are selected as predictors for the answers to each of the 16 items of Section D of survey Q3.

As alternative methods to select the features, we also tested the Minimum Redundancy Maximum Relevance (MRMR) and ReliefF algorithms [1, 2]. However, the MRMR algorithm yielded worse predictive power for our classifier. For example, with respect to the traumatic symptom "Recurrent thoughts or memories of the most hurtful or terrifying events", the MRMR algorithm selects features 2, and 5 to 9 of survey Q3, section D, and the best model (the SVM – Medium Gaussian with modified cost function) has inferior performance (accuracy 0.78, sensitivity 0.83, fall-out 0.19, AUC = 0.84) compared to those reported in Table 7 of the main text. As for the predictors of "Feeling jumpy, easily startled" and "Suddent emotional or physical reaction when reminded of the most hurtful events when reminded of trauma", the MRMR algorithm only selected features 5 and 9 of survey Q3, section D, but the corresponding model failed to meet the minimum criteria on the performance metrics we selected.

As for the ReliefF algorithm, we explored the effect of the number k of nearest neighbors of each observation on the relevant selected features. As shown in Table 2, the algorithm was too sensitive to the selection of its key parameter, and therefore we elected not to use it.

Supplementary Table 1. For each of the 16 items of survey Q3, section D, we report the features of survey Q3, section C, selected as predictors using a χ^2-test.

Item	*Features*
1	2, 5, 7, 8
2	5, 7, 8
3	5, 7, 8
4	2, 3, 5, 8
5	5, 8
6	3, 5, 7, 8, 9
7	3, 4, 5, 7, 8
8	1, 5, 7, 8
9	5, 7
10	4, 5, 8
11	3, 4, 5, 9
12	5, 9
13	5, 6
14	2, 5, 6, 7, 8
15	2, 3, 4, 5, 7, 8, 9
16	2, 4, 5, 7, 8, 9

Supplementary References

1. Chris Ding and Hanchuan Peng. Minimum redundancy feature selection from microarray gene expression data. *Journal of Bioinformatics and Computational Biology*, 3(02):185–205, 2005.

2. Ryan J Urbanowicz, Melissa Meeker, William La Cava, Randal S Olson, and Jason H Moore. Relief-based feature selection: Introduction and review. *Journal of Biomedical Informatics*, 85:189–203, 2018.

Supplementary Table 2. For each of the 16 items of survey Q3, section D, we report the features of survey Q3, section C, selected as predictors using by the ReliefF algorithm as a function of the number of nearest neighbors k.

	Number of neighbors k				
Item	8	9	10	11	12
1	1, 2, 5, 7, 8, 9	1, 2, 5, 7, 8, 9	2, 5, 7, 8, 9	1, 2, 5, 7, 8, 9	2, 5, 7, 8, 9
2	1, 2, 3, 4, 5, 6, 7	1, 2, 3, 4, 5, 6, 7, 9	1, 3, 4, 5, 6, 7	1, 3, 4, 6, 7	1, 3, 4, 6, 7, 9
3	1, 2, 4, 5, 6, 7, 8, 9	1, 4, 5, 6, 7, 8, 9	1, 4, 5, 6, 7, 8, 9	1, 4, 5, 6, 7, 8, 9	4, 5, 6, 7, 8, 9
4	2, 4, 6, 7, 9	1, 5, 6, 7, 9	1, 5, 6, 7, 9	6, 7, 9	6, 9
5	4, 6, 7, 9	6, 7, 9	6, 7, 9	6, 7, 9	6, 7, 9
6	1, 2, 4, 5, 6, 9	1, 4, 5, 6, 9	1, 4, 5, 6, 9	1, 4, 5, 6, 9	4, 5, 6, 9
7	1, 2, 5, 6, 7, 9	1, 5, 6, 7, 9	1, 2, 5, 6, 7, 9	1, 2, 5, 6, 7, 9	1, 2, 4, 5, 6, 7, 9
8	1, 4, 5, 7, 8, 9	1, 4, 5, 7, 8, 9	1, 4, 5, 7, 8, 9	1, 5, 7, 8, 9	1, 4, 5, 7, 8, 9
9	5, 6, 7, 9	5, 6, 7, 9	1, 5, 6, 7, 9	5, 6, 7, 9	5, 6, 7, 9
10	2, 5, 6, 7, 8, 9	5, 6, 7, 8, 9	5, 6, 7, 8, 9	5, 6, 7, 9	5, 6, 7, 9
11	1, 2, 3, 4, 5, 6, 7, 9	1, 2, 3, 4, 5, 6, 7, 9	1, 2, 3, 4, 5, 6, 7, 9	1, 2, 3, 4, 6, 7, 9	1, 2, 3, 4, 6, 7, 9
12	2, 3, 4, 6, 8, 9	3, 4, 6, 7, 8, 9	3, 4, 6, 7, 8, 9	3, 4, 6, 7, 8, 9	3, 4, 6, 7, 9
13	1, 3, 4, 6, 9	1, 3, 4, 5, 6, 7, 9	1, 3, 4, 5, 6, 7, 9	1, 4, 5, 6, 7, 9	4, 5, 6, 7, 9
14	1, 2, 5, 6, 7	1, 5, 6, 7	1, 5, 7, 8	1, 5, 6, 7	1, 5, 6, 7
15	2, 4, 5, 6, 7, 9	3, 4, 5, 6, 7, 8, 9	3, 4, 5, 6, 7, 8, 9	3, 4, 5, 6, 7, 9	3, 4, 5, 6, 7, 9
16	1, 2, 4, 5, 6, 9	1, 2, 4, 5, 6, 9	1, 2, 4, 5, 9	1, 4, 5, 6, 9	1, 2, 4, 5, 6, 9

Supplementary Table 3. List of the classification algorithms that have been trained and tested, sorted by family. Only the algorithms with the best performance have been reported in the main text.

Family	*Algorithm*	
Tree	– Coarse	– Fine
	– Medium	
SVM	– Linear	– Quadratic
	– Cubic	– CoarseGaussian
	– MediumGaussian	– FineGaussian
kNN	– Fine	– Medium
	– Coarse	– Cosine
	– Cubic	– Weighted
Ensemble	– Bag	– Boost
	– RUSBoost	– SubSpaceDiscr
	– SubSpaceKNN	
